# Conformational stabilization as a strategy to prevent nucleophosmin mislocalization in leukemia

**DOI:** 10.1038/s41598-017-14497-4

**Published:** 2017-10-24

**Authors:** María A. Urbaneja, Lars Skjærven, Oscar Aubi, Jarl Underhaug, David J. López, Igor Arregi, Marián Alonso-Mariño, Andoni Cuevas, José A. Rodríguez, Aurora Martinez, Sonia Bañuelos

**Affiliations:** 1 Biofisika Institute (UPV/EHU, CSIC) and Department of Biochemistry and Molecular Biology, University of the Basque Country (UPV/EHU), Leioa, Spain; 2 Department of Biomedicine, University of Bergen, Bergen, Norway; 3 Department of Genetics, Physical Anthropology and Animal Physiology, University of the Basque Country (UPV/EHU), Leioa, Spain; 4 K.G. Jebsen Centre for Neuropsychiatric Disorders, University of Bergen, Bergen, Norway; 5 Present Address: Department of Chemistry, University of Bergen, Bergen, Norway; 6 R&D Department, Roxall España, Bilbao, Spain

## Abstract

Nucleophosmin (NPM) is a nucleolar protein involved in ribosome assembly and cell homeostasis. Mutations in the C-terminal domain of NPM that impair native folding and localization are associated with acute myeloid leukemia (AML). We have performed a high-throughput screening searching for compounds that stabilize the C-terminal domain. We identified three hit compounds which show the ability to increase the thermal stability of both the C-terminal domain as well as full-length NPM. The best hit also seemed to favor folding of an AML-like mutant. Computational pocket identification and molecular docking support a stabilization mechanism based on binding of the phenyl/benzene group of the compounds to a particular hydrophobic pocket and additional polar interactions with solvent-accessible residues. Since these results indicate a chaperoning potential of our candidate hits, we tested their effect on the subcellular localization of AML-like mutants. Two compounds partially alleviated the aggregation and restored nucleolar localization of misfolded mutants. The identified hits appear promising as pharmacological chaperones aimed at therapies for AML based on conformational stabilization of NPM.

## Introduction

Nucleophosmin (NPM), a protein normally enriched in cell nucleoli, is in charge of several functions that affect cell homeostasis^1,2^. Among them, NPM assists in ribosomal biogenesis, modulates the stability of tumor suppressors such as p53 and ARF, is involved in the control of centrosome duplication and participates in DNA repair processes^1,2^. NPM is a pentameric protein that consists of several domains. Each subunit contains a β-structured^3^ oligomerization domain of ca. 125 residues that forms the compact core and is connected through a long (125 aa) and flexible linker to the small (50 aa), globular, α-helical C-terminal domain^4^. NPM behaves as a nucleolar hub, interacting with many protein partners, as well as nucleic acids^5^. The binding to G-rich DNA and/or RNA involves the C-terminal domain^6^ which is probably responsible for the protein retention in nucleoli^7^. Although mostly nucleolar, NPM continuously shuttles between cytoplasm, nucleoplasm and nucleoli to perform its functions^8^. This traffic is mediated by importin α/β and CRM1 transport receptors^9^.

Dysfunction of NPM can lead to cancer pathologies^2,10^. In particular, *NPM1* is the most frequently mutated gene in acute myeloid leukemia (AML)^11^. Mutations correlate with the aberrant cytoplasmic localization of NPM in blasts from the patients^11^. This mislocalization of NPM is a hallmark of this subtype of AML, termed “NPMc + ” on that basis, and seems to be a driver event in the development of the disease^12^.

The AML-associated NPM mutations^11^ involve frame shift insertions of few bases at the end of the *NPM1* gene, resulting in a mutant protein with an abnormal sequence in the C-terminal 9–11 residues. The altered sequence implies the loss of one (Trp290, as in NPM mutant E) or more frequently two (Trp288 and Trp290, as in NPM mutant A) tryptophan residues^11^ that are essential for the packing of the hydrophobic core of the C-terminal domain, and therefore, this domain is unable to fold properly in mutated NPM^4^. The C-terminal domain of mutant A has been shown to completely lack any secondary structure, while mutant E keeps a partly folded structure^4^. The defective folding of the C-terminal domain results in the inability of NPM to bind G-rich sequences^7,13^, and consequently to be retained in the nucleolus^4,7^. In addition, the mutated C-terminal sequence functions as a novel nuclear export sequence (NES)^14^, that adds to the intrinsic, weak NES or NESs of NPM^9^, directing the exacerbated export of the protein by CRM1. Both factors, unfolding of the C-terminal domain and acquisition of a novel NES, have been shown to jointly contribute to the aberrant cytoplasmic accumulation of mutant NPM^14,15^.

Standard chemotherapy provides complete cure of AML in only about 30% of patients and, therefore, alternative pharmacological strategies are of interest. In this regard, NPM represents an attractive target for therapy^16^. Due to the alterations described above, NPMc + AML can be considered a misfolding disease, which could be therapeutically addressed by using pharmacological chaperones, small molecules that bind to the mutated protein, increasing its conformational stability. As recently reviewed^17^, there are now several compounds with pharmacological chaperone potential for a large number of misfolding diseases that have been successfully tested in animal models, and a few that are already in phase II and III clinical trials. These compounds may restore the native fold and/or the proper localization of the mutants^18^. The search for pharmacological chaperones can be achieved through high-throughput screening (HTS) of compound libraries using differential scanning fluorimetry (DSF) or other methods monitoring increase of stability in the target protein^19,20^. This approach has been successfully applied to search for drugs stabilizing various misfolding mutants of enzymes involved in metabolic disorders^20,21^.

Restoration of the native fold of mutant NPM would be expected to favor binding to nucleolar ligands and therefore increase retention of NPM in the nucleoli. The fact that reinsertion of W288 and W290, key elements for the folding of the C-terminal domain, within the sequence context of mutant A relocates the protein to the nucleoli^14^, further supports this notion. We have performed a HTS for compounds that increase the midpoint melting temperature (*T*_m_) of the C-terminal domain of NPM. Several hits were subsequently validated based on their ability to bind and stabilize full length NPM and a C-terminal domain carrying AML-linked mutations, and their effect on human cells expressing YFP-tagged AML-related NPM mutants has been assessed. Structural bioinformatics analyses including pocket identification and molecular docking complemented with molecular dynamics (MD) simulations support a molecular mechanism of correction by these hits based on the stabilization of the hydrophobic core of NPM C-terminal domain. Our results have identified two compounds that are able to favorably modulate the dysfunction of AML mutants.

## Results and Discussion

### High-throughput screening by differential scanning fluorimetry (DSF)

**Figure 1:**
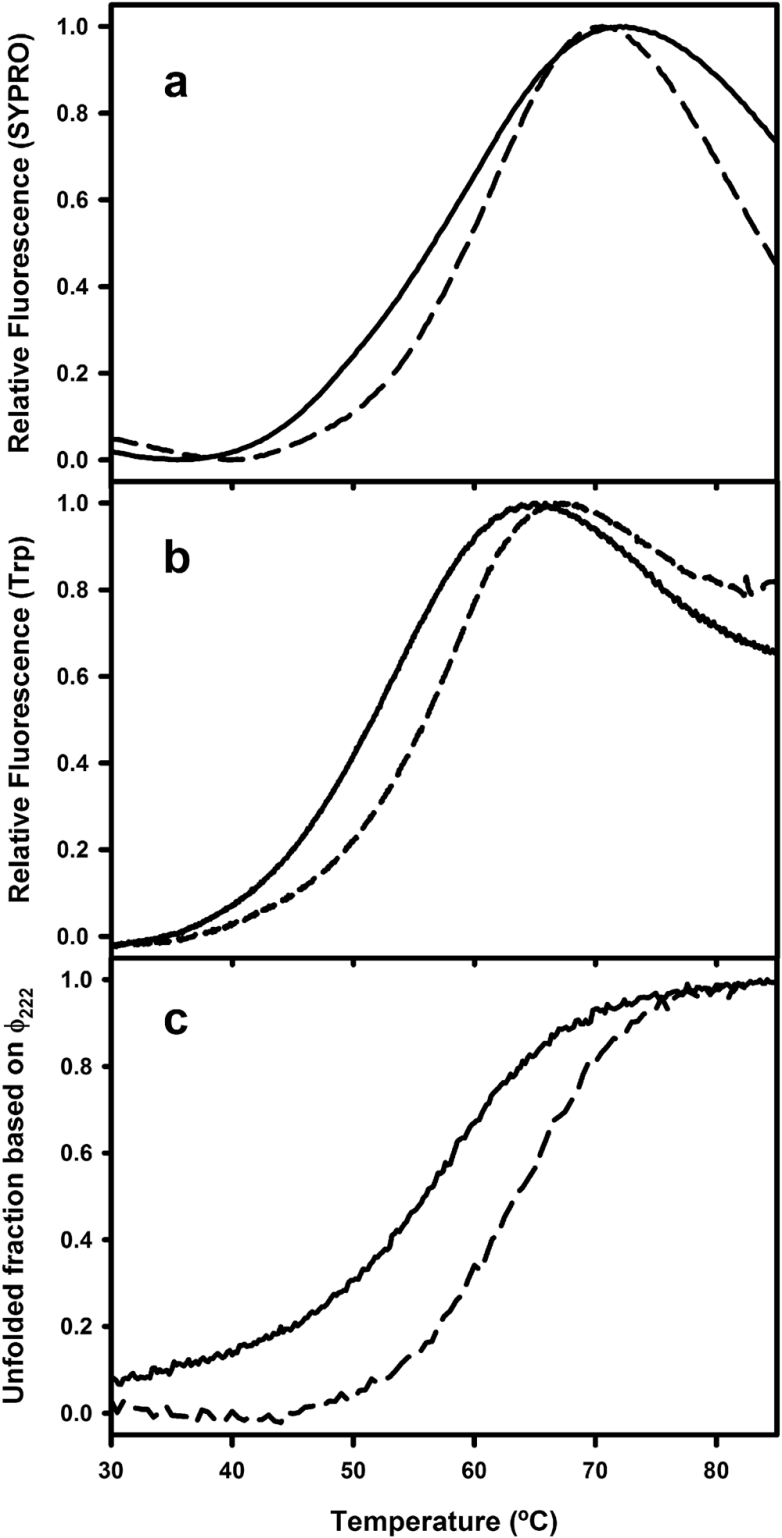
Thermal stability of full length nucleophosmin (NPM) and isolated C-terminal domain. Thermal denaturation profiles monitored by DSF (**a**), tryptophan emission fluorescence (**b**) and circular dichroism (ellipticity at 222 nm) (**c**). Solid and dashed lines correspond to the isolated C-terminal domain and to full length NPM, respectively. The protein concentrations were 10 µM for full-length NPM and 50 µM for the C-terminal domain, prepared in 50 mM sodium phosphate buffer, pH 7.5.

The NPM C-terminal domain displays a relatively autonomous thermal transition, albeit modulated by the rest of the protein. DSF (Fig. 1a), intrinsic tryptophan fluorescence (Fig. 1b), and circular dichroism (CD) at 222 nm (Fig. 1c) provided *T*_m_-values of 56.8 ± 0.2 °C, 52.2 ± 0.4 °C, and 57 ± 1 °C, respectively, for C-terminal domain and 59.6 ± 0.1 °C, 56 ± 0.4 °C, and 63.5 ± 1 °C, respectively, for full-length NPM. Thus, although monitoring somehow different events –reflecting rather unfolding of the tertiary (DSF and Trp fluorescence) or secondary (CD) structures– the three methods confirm, in line with reported results^22^, that the C-terminal domain of recombinant NPM is slightly less stable in isolation than in the context of the full length protein.

**Figure 2:**
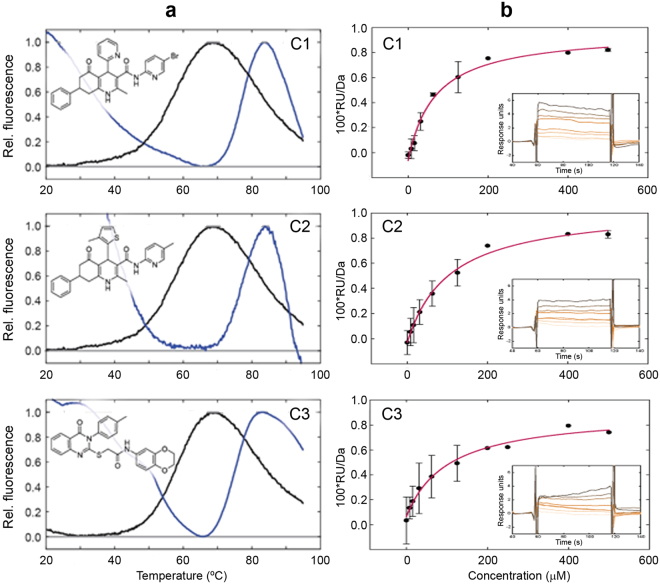
Hit compounds C1, C2 and C3 bind NPM, stabilizing the C-terminal domain. DSF profiles of NPM C-terminal domain in the absence (black line) and presence (blue line) of hit compounds C1, C2 and C3, and chemical formulae of the compounds (**a**). Isotherms for compound hits (C1, C2 and C3) binding to NPM analyzed by surface plasmon resonance (**b**). Experiments were performed in triplicate with each inset depicting a representative original sensorgram at compound concentrations 0, 7.81, 15.6, 31.2, 62.5, 125, 250 and 500 µM (corresponding increase in color intensity), all in 5% DMSO. *S*_0.5_ values of 68 ± 13 µM for C1, 105 ± 17 µM for C2 and 102 ± 23 µM for C3 were obtained from fittings to a 1:1 binding model.

Since the most recurrent mutations A and E affect the packing of the hydrophobic core of the C-terminal domain, and having in mind that we are looking for compounds that would specifically bind to that domain, we performed a DSF-based HTS with the isolated C-terminal domain (residues 243–294), screening a 2560 compound subset of the MyriaScreen diversity collection. The *T*_m_ value for the C-terminal domain of NPM in the presence of each compound was compared to the value for the control (with 4% DMSO) to determine the up-shift in *T*_m_ (∆*T*_m_). After removal of PAINS^23^, we reached a final list of 25 hit compounds that stabilized the domain with Δ*T*_m_ > 6 °C, and the top-three, hereafter termed C1, C2 and C3, displayed the largest Δ*T*_m_ ( > 17 °C) when tested at ~200 µM (Fig. 2a and Table S1). C1 and C2, but not C3, show high chemical similarity, and both share a common scaffold with two fused heterocycles and a pyridine ring (“a” in Figure S1). Based on Δ*T*_m_ compounds C1, C2 and C3 were selected for further investigations.

### Binding validation and stabilization effect of identified hits on full-length NPM

We further confirmed by SPR experiments that the top three hit compounds also bind to full-length NPM (Fig. 2b). Response units from concentration-dependent steady state measurements were analyzed assuming a 1:1 binding model, providing *S*_0.5_ values of 68 ± 13 µM for C1, 105 ± 17 µM for C2 and 102 ± 23 µM for C3.

We further used isothermal fluorescence experiments to validate a stabilizing effect of the top-hit C1 on full length NPM. As seen in the time course of the change in Trp emission fluorescence upon unfolding of the protein, addition of C1 significantly delayed the unfolding transition (Figure S2). Of note, the Trp fluorescence is specific of the C-terminal domain (where the unique two Trp residues of NPM are located), indicating that this domain is the specific target of the stabilization.

**Figure 3:**
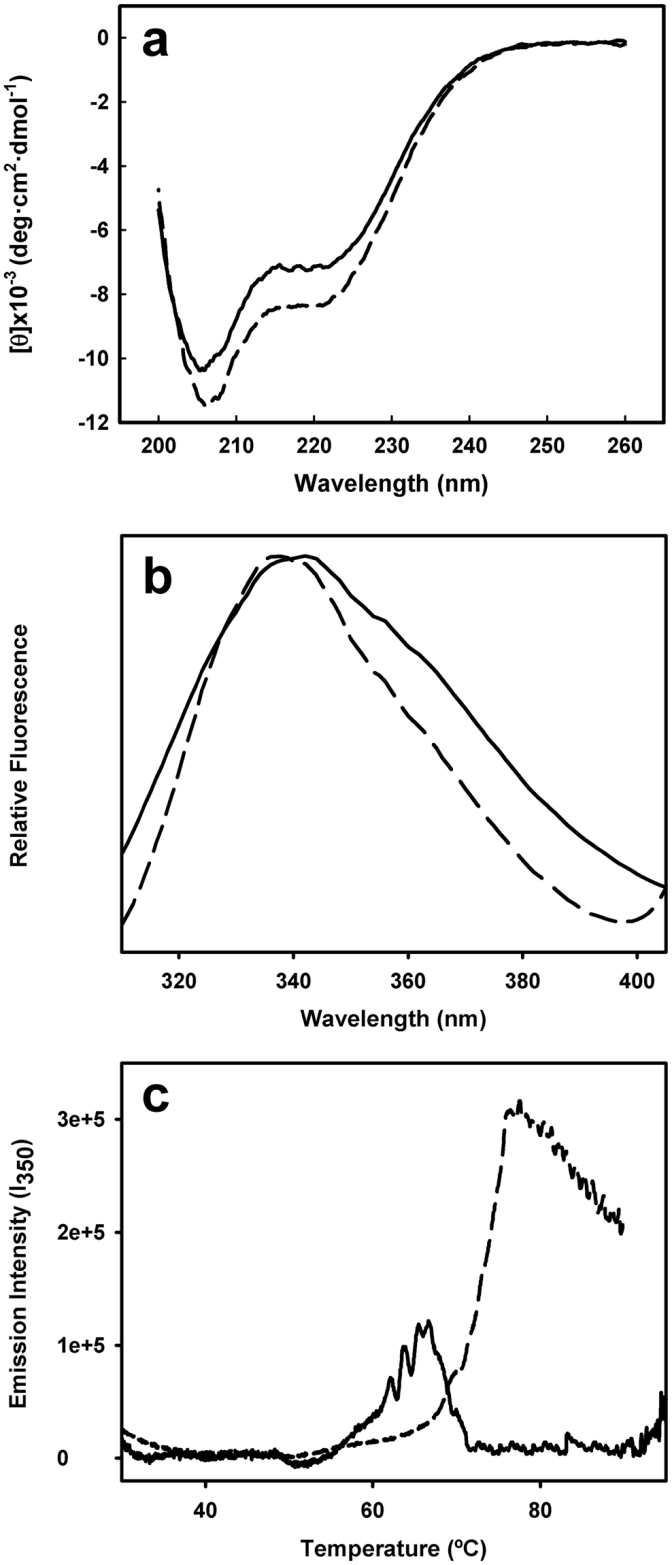
Effects of C1 on circular dichroism, fluorescence emission and thermal behavior of NPM mutant E C-terminal domain. A synthetic peptide corresponding to the C-terminal domain of mutant E was diluted in 50 mM phosphate buffer (pH 7.5) to a final concentration of 50 μM. Circular dichroism spectra (**a**), Trp fluorescence emission spectra (**b**) and thermal scans (**c**) were recorded in the absence (solid line) and presence (dashed line) of 150 µM C1. For CD experiments (**a**), C1 was prepared in ethanol, whereas for fluorescence spectroscopy (**b**) and (**c**), C1 was prepared in DMSO. In all cases, the final concentration of solvent was 1.5% (v/v).

With the aim of testing the effect of the compounds on AML-related NPM mutants, we produced recombinant mutant A (mutA) and E (mutE), both full-length and fragments corresponding to the C-terminal region. The instability of these mutant forms, however, led to partially degraded preparations, not suitable for biophysical characterization. We thus used a synthesized peptide corresponding to the mutE C-terminal domain. This mutant domain displayed a far-UV CD spectrum compatible with the presence of α-helix secondary structure (Fig. 3a), which agrees with the partially folded structure described elsewhere^4^. Addition of C1 induced a slightly more negative ellipticity (Fig. 3a), compatible with an increase in α-helical content. We then investigated the effect of C1 on the conformational properties of the domain by fluorescence spectroscopy. In the presence of C1 the emission spectrum displayed a moderate shift to lower wavelengths (from 341 to 338 nm) (Fig. 3b), suggesting that Trp288, partially exposed to the solvent, senses a less polar environment. This blue shift might be caused by a direct contact with the compound or/and to a C1-folding aid effect. Furthermore, the mutant domain exhibited a much more significant and cooperative thermal transition in the presence of C1, which is in addition displaced to higher temperatures (Δ*T*_m_ ~ 10 °C) (Fig. 3c). Due to the fluorescence properties of C3, no conclusive results could be obtained on the effects of that compound. These fluorescence results thus suggest that the stabilizing effect induced by C1 binding might be the result of a C1-mediated refolding and/or stabilization event, which would lead to a more compact folding of the C-terminal domain of NPM mutant E.

### Computational prediction of C1 binding to the C-terminal domain of NPM

**Figure 4:**
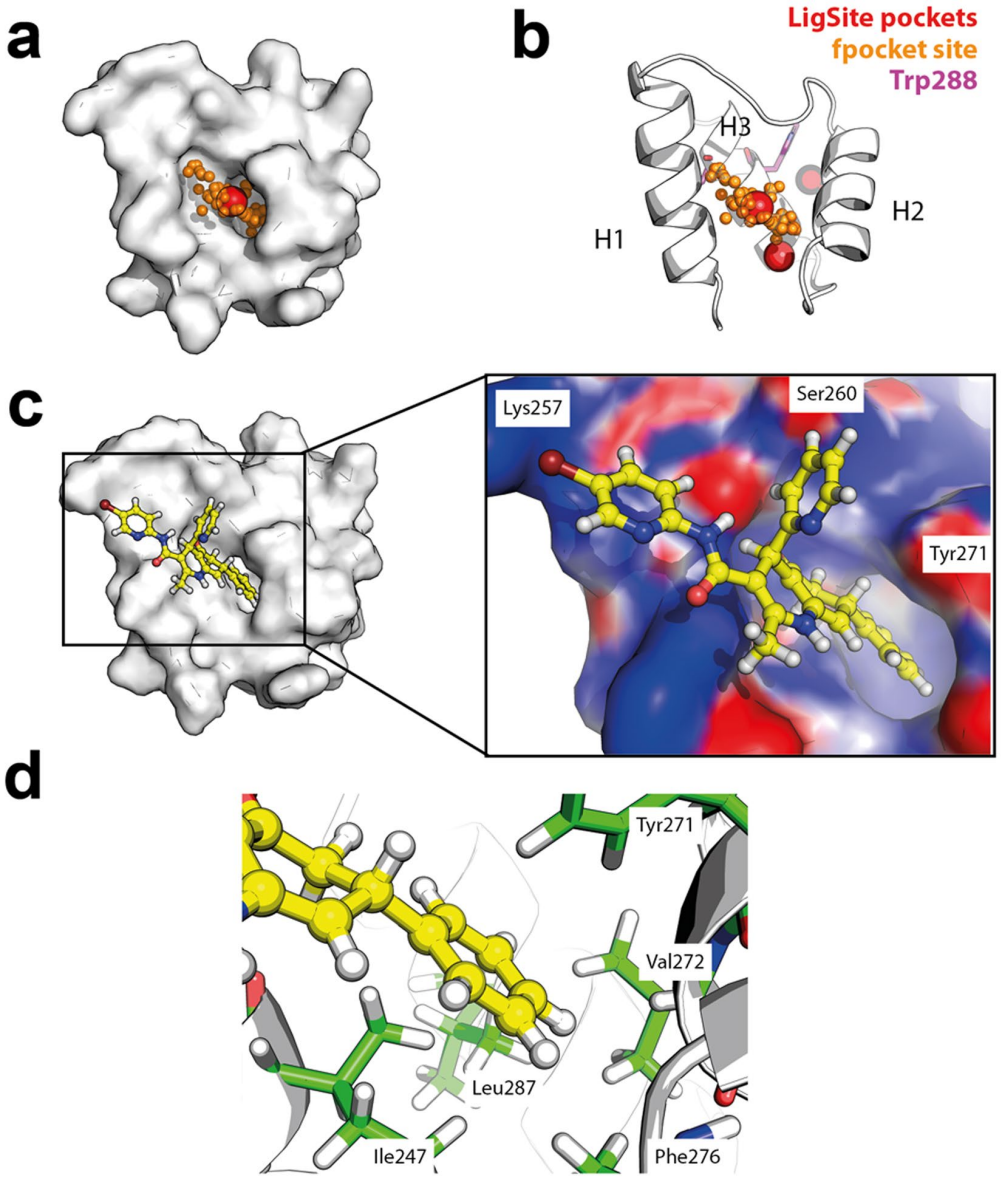
Prediction of C1 binding to the C-terminal domain of NPM. Structure of the C-terminal domain of NPM (PDB ID: 2LLH) is shown in surface (**a**) and cartoon representation (**b**) with binding pockets predicted using fpocket (orange) and LigSite (red). Binding mode as predicted by computational protein-ligand docking (**c**,**d**) suggests that the aromatic phenyl ring of C1 occupies the hydrophobic pocket presented in (**a**), and also establishes favorable polar interactions with solvent accessible residues. A close-up view of C1 in the pocket is shown in (**c**), with the surface of the domain coloured according to electrostatic surface potential, with charge ranging from -3 kT/e (deep red) to 3 kT/e (deep blue).

To predict the mode of binding of C1 to NPM we probed the 20 conformations of the previously released NMR structure of the C-terminal domain of NPM^24^ (PDB ID 2LLH) for pockets on the protein surface. Two pockets were identified with a good druggability score ( > 0.9), as assessed by both the fpocket^25^ and LigSite algorithms^26^. The highest scoring pocket (pocket #1), located between helices H2 and H3, was visible in multiple conformers of the C-terminal domain of NPM; another one (pocket #2), scoring second and located between H1 and H2, showed a deep hydrophobic cavity protruding into the interior of the protein (Fig. 4a,b). Computational protein-ligand docking of C1 to all 20 NMR conformers, using the entire domain as possible docking site, predicts pocket #2 as the one with the best capability to accommodate C1. This is indicated by the favorable docking score compared to the other sites present in all the 20 NMR conformers (Figure S3). In the docked structure, the phenyl ring of C1 occupies the hydrophobic cavity detected by the pocket programs (Fig. 4c), with a number of hydrophobic residues flanking the cavity (Fig. 4d). One of these residues, Leu287, is adjacent to residues Trp288 and 290, affected by AML mutations. C1 also establishes favorable polar interactions with Lys257, Ser260 and Tyr271 (Fig. 4c). A 200 ns long molecular dynamics simulation of the C-terminal domain:C1 complex was subsequently conducted to investigate the conformational stability of the predicted binding mode. The simulation showed that C1 maintains its initial binding mode throughout the simulation with the phenyl ring buried in the hydrophobic pocket (Figure S4).

**Figure 5:**
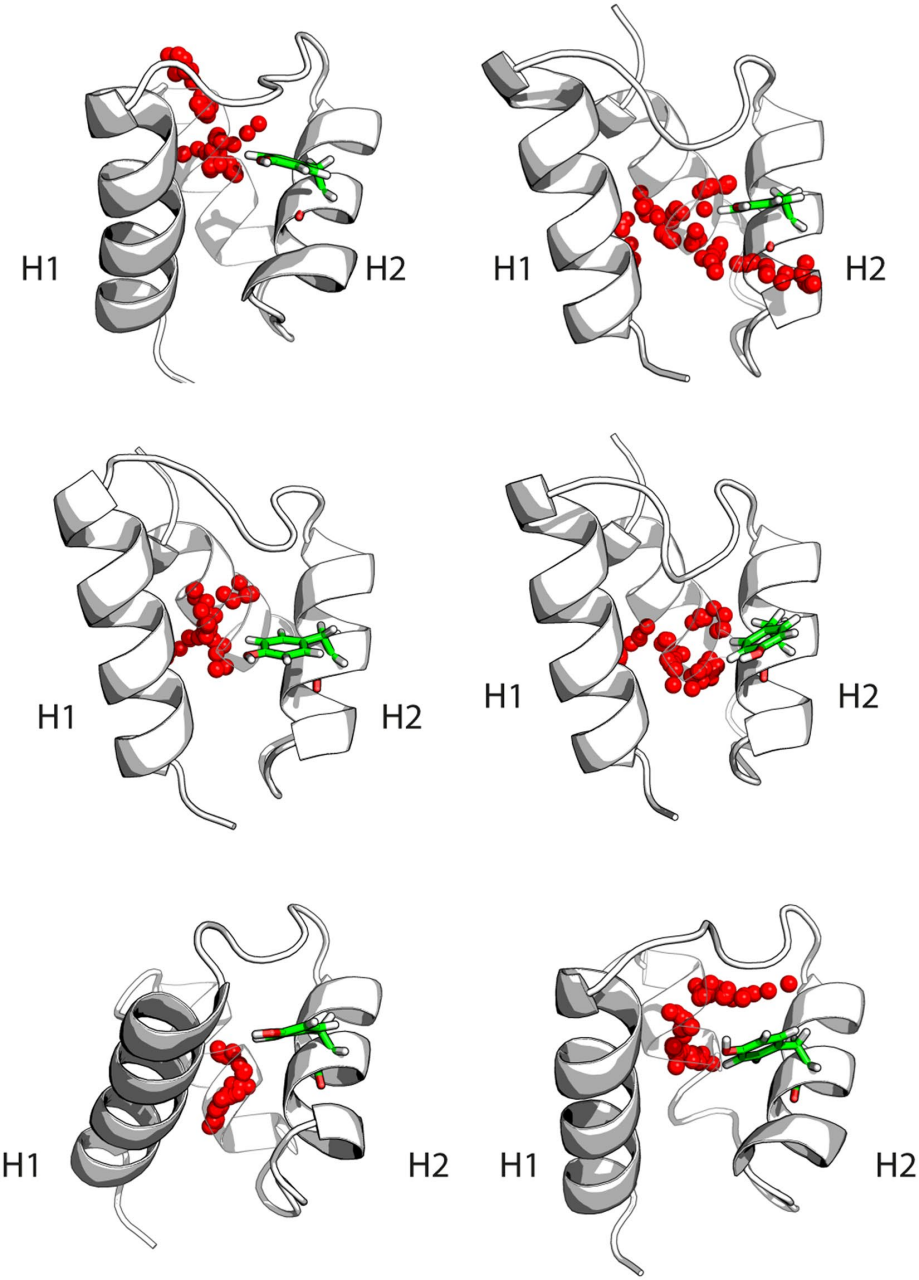
Computational pocket identification in conformers generated by molecular dynamics of NPM mutant E C-terminal domain. MD generated conformers with the top scoring pockets as assessed by fpocket druggability score are shown (pocket #2 only). Each panel shows a MD conformer in cartoon representation with the predicted pocket shown in red spheres. Tyr271 is depicted in sticks representation (green).

To explore the presence of pocket #2 in the mutant E variant of NPM C-terminal domain, we performed 200 ns long MD simulations of mutant models generated by homology modeling from 4 representative NMR structures. Pocket analysis with fpocket was subsequently performed over the resulting MD trajectories. All identified pockets were filtered on distance to pocket 2 and finally sorted on druggability score. The results clearly showed the presence of pocket 2 also for the mutant E model variant of the domain (Fig. 5). Our results thus point to this druggable pocket as the probable binding site of C1 also for the mutant variant, as supported by the large binding-induced stabilization observed by Trp-fluorescence results (Fig. 3b). Comparative analyses with the possible binding modes of C2 and C3 support that these hits also may bind at the same pocket as C1 does. Accordingly, the 2-methyl (C2) instead of Br (C1) substitution on the pyridine ring (Figure S1) would lead to the loss of the favorable polar interaction with Lys257, explaining the reduction in affinity for C2. For C3 the benzene ring in the oxoquinazolin group might occupy the position of the phenyl group in C1 whereas the rest of the molecule maintains the polar interactions with the protein.

All together our results point to the large thermal stabilization provided by these compounds (ΔT_m_ > 17 °C) being associated to the “covering” of a solvent accessible hydrophobic area, which represent a good druggable pocket in NPM. Interestingly top-hit C1 establishes in addition favorable polar interactions with solvent-accessible residues that may further stealth and stabilize the hydrophobic core.

### Effect of C1, C2 and C3 compounds on the subcellular localization and aggregation state of mutant NPM

**Figure 6:**
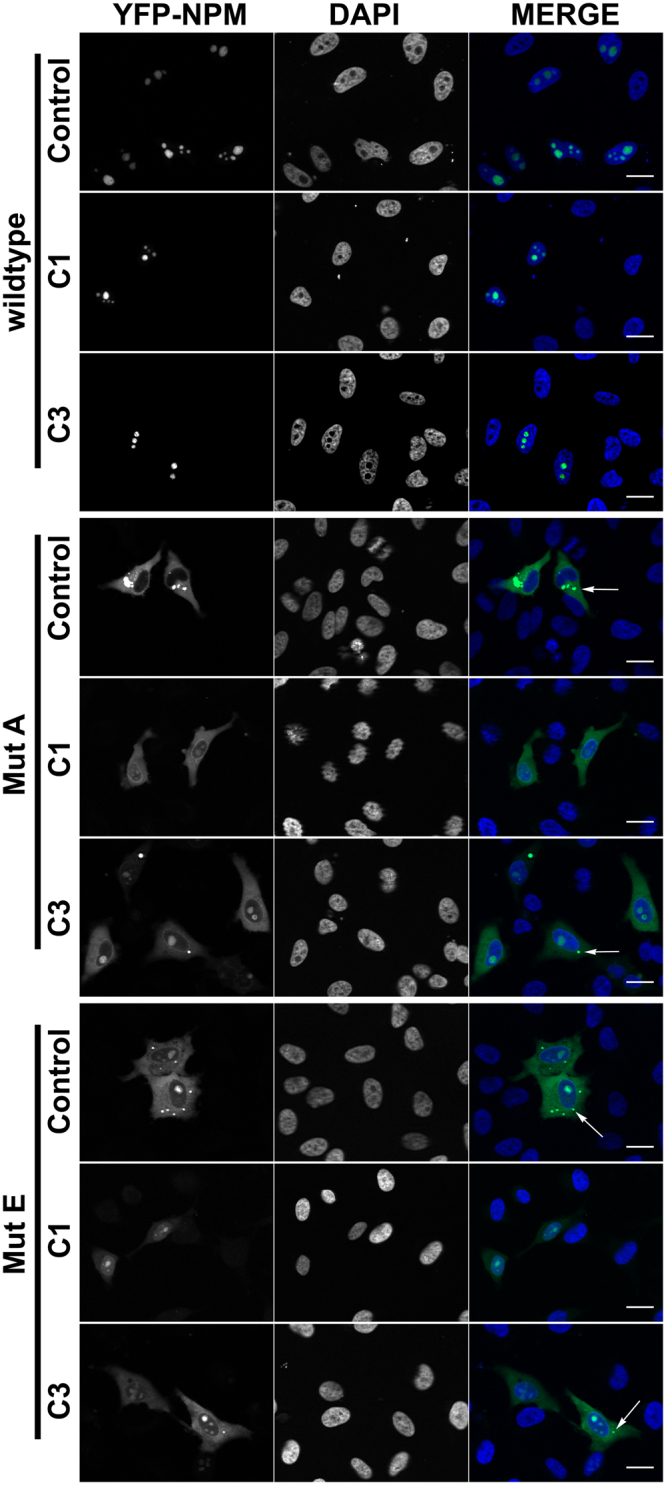
Subcellular localization of YFP-NPM (wild type, mutant E or mutant A) in examples of untreated HeLa cells or treated with compounds C1 and C3. Confocal microscopy images of HeLa cells in the absence or presence of 50 μM C1 or C3. YFP fluorescence is shown in green and DNA, stained with DAPI, in blue. Bright green fluorescence dots in the cytoplasm of cells transfected with the mutants are assigned to protein aggregates (arrows, see text).

We then probed the hit compounds in a cellular setting, investigating their possible effects on the subcellular localization of NPM mutants. HeLa cells were transiently transfected with the different YFP-NPM constructs (wild type, mutant A, mutant E), and treated with compounds C1, C2 and C3. In the absence of compounds, cells transfected with wild type YFP-NPM displayed bright fluorescence in nucleoli (Fig. 6). The nucleolar localization was confirmed by immunostaining with the nucleolar marker fibrillarin (Figure S5). By contrast, mutant E showed a more diffuse fluorescence throughout the cell, locating mostly in the nucleoli of some cells, but rather in nucleoplasm and/or cytoplasm, in others. The distribution of mutant A was even more cytoplasmic. This observed localization of NPM mutants A and E was reverted upon treatment with the specific inhibitor leptomycin B (LMB) (not shown), indicating that their export is mediated by CRM1, as previously described^9,27^. Similar results were obtained with the HEK293T cell line (not shown). The observed behavior agrees with previously published results using ectopically expressed fluorescent protein-NPM constructs^27,28^, and reproduces the partially cytoplasmic localization of NPM in blasts of AML patients^29^, suggesting that this system is a valid model of the localization dynamics of NPM in AML. Additionally, and only in cells expressing mutant variants of YFP-NPM, we observed the presence of bright fluorescent dots, which we attributed to protein aggregation, probably due to the defective folding of the mutant proteins (Fig. 6). In agreement with this observation, aggregation-prone amyloidogenic regions were recently described in NPM C-terminal domain^30^.

The aggregation seems more severe in cells transfected with mutant E than in those expressing mutant A, even though the latter is expected to be more structurally affected^4^ and hence more aggregated^31^. On the other hand, those cells expressing low amounts of mutant (A or E) YFP-NPM displayed less aggregates and relatively more nucleolar NPM staining. This could be due to the influence of endogenous, wild type NPM, expected to form mixed oligomers with the mutant protein^28^. Nevertheless, the aggregation cannot exclusively be ascribed to excessive overexpression of the constructs, since the exogenous:endogenous NPM ratio normalized by the transfection efficiency did not correlate with the presence of aggregates. Thus YFP-NPM mutE, displaying the highest density of aggregates, was expressed in lower amounts compared to other constructs (Figure S6).

**Figure 7:**
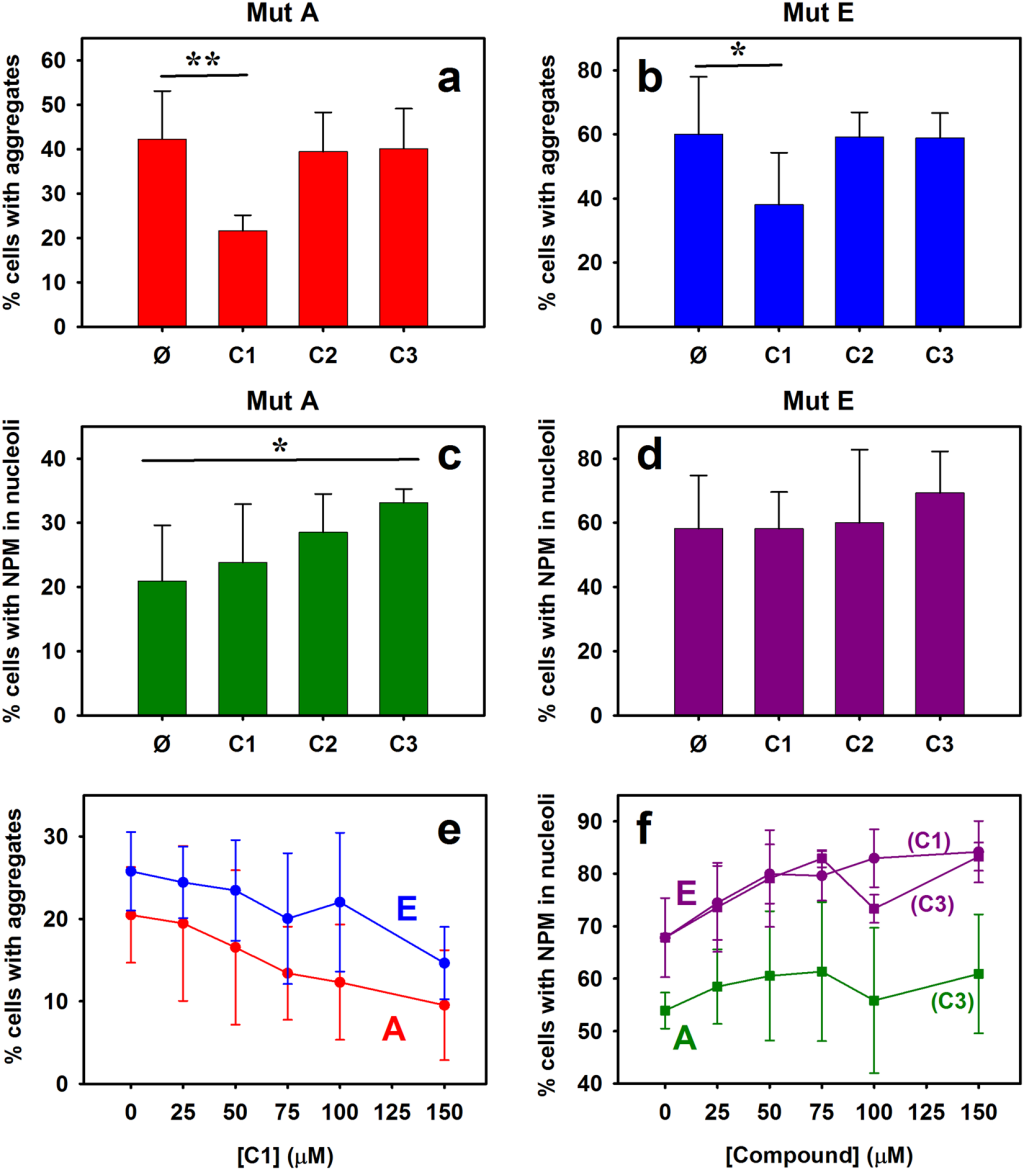
Effect of treatments with the compounds on HeLa cells transfected with NPM mutants. Percentage of cells containing aggregates (**a** and **b**) and displaying maximum YFP intensity in the nucleoli (**c** and **d**), for mutant A (**a** and **c**) and mutant E (**b** and **d**). Values shown are averages of at least 4 experiments ( ≥ 200 cells per experiment), and the corresponding SD (*P < 0.05; **P < 0.01). Aggregation (**e**) and nucleolar localization (**f**) of NPM mutants as a function of compounds concentration. Data for C1 are displayed with circles and for C3 with squares.

We explored the effect of the selected compounds on NPM behavior. Upon treatment of HeLa cells with 50 µM either C1, C2 or C3, a fraction of the cells treated with C1 and C3 seemed to display less aggregated and/or more nucleolar distribution of mutant NPM as compared to untreated cells. Examples of cells showing the discussed effects are shown in Fig. 6. Semiquantitative evaluation of nucleolar localization and aggregation degree was performed in more than 200 cells (including cells showing different expression levels of YFP-NPM constructs) per condition, confirming statistically significant effects of C1 and C3 on YFP-NPM behavior compared to the corresponding untreated controls (Fig. 7). Microscopy images were blind-analysed (in which the identity of the samples was hidden) to have an unbiased scoring for the presence of aggregates and nucleolar fluorescence. C1 reduced protein aggregation of both mutants (Fig. 7a,b), whereas C3 seemed to favor their nucleolar retention (Fig. 7c,d). The lack of effect of C2 is most probably associated to its lower affinity for NPM (see above; Fig. 2b), and it further supports the relation between the affinity and stabilizing effect *in vitro* with the in-cell chaperone effect observed for C1 and C3.

In order to corroborate the effect of the compounds on the behavior of mutant NPMs, we evaluated the concentration dependence of their activities. The titration experiments showed that aggregation of both mutants A and E was indeed reduced upon addition of C1, in agreement with the results obtained at 50 μM of the compound, and that this effect correlates with the compound concentration (Fig. 7e). In addition, an ability to relocate mutant E was also revealed for this compound (from 68 ± 7% of cells with maximum fluorescence in nucleoli in the absence of the compound to 84 ± 6% in the presence of 150 μM C1) (Fig. 7f). Similarly, compound C3 favored nucleolar localization of both mutants in a concentration-dependent manner (Fig. 7f). Importantly, the global effects of the compounds (i.e. less aggregation, more nucleolar localization) were generally found when blindly evaluated by two independent observers (Fig. 7a–d vs. e,f), supporting the reliability of the results.

Some of the observed effects showed saturation at 50–100 μM, which can be due, in part, to toxicity of the compounds. To evaluate this possibility, we carried out sulforhodamyne B-based (SRB) toxicity assays and found that the hit compounds, in particular C1, reduced cell viability with estimated IC_50_ values of approximately 50 μM (data not shown). Interestingly, the concentration initially chosen (50 μM) seems to be optimal for getting the desired effects without excessive toxicity. Given the compounds toxicity, their effect on NPM localization / aggregation might be related to a stress-induced proteostatic response. We checked that this was unlikely, however, since the amount of the stress marker Hsp70, based on western blot, was not significantly altered upon treatment with the compounds (Figure S7). This result suggests that the compounds exert *bona fide* effects acting directly on NPM.

Collectively our cellular experiments indicate that the compounds C1 and C3 are able to favor the nucleolar retention of NPM mutants and reduce their aggregation. Recently, two compounds, oridonin^32^ and avrainvillamide^33,34^ have been described to be able to promote NPMc + nucleolar re-localization. Although the molecular basis of the effect of these drugs is so far unclear, it was suggested that the binding of avrainvillamide to both NPM and CRM1 might inhibit NPM export. Our hit compounds, on the other hand, seem to have a different mechanism of action as pharmacological chaperones, largely increasing the conformational stability of NPM through binding to the C-terminal domain. Both C1 and C3 are Lipinski compounds, and neither of them is classified as PAINS^23^. These hits possess a chemical structure that may be subjected to hit-to-lead expansion and optimization through a medicinal chemistry campaign to increase their affinity for NPM and thus their potency in cells, improve their ADME/PK (absorption, distribution, metabolism, excretion and pharmacokinetics) and toxicity profile.

## Conclusion

Through HTS we have identified three compounds that stabilize the C-terminal domain of NPM. Two of them, C1 and C3, have subsequently been validated for pharmacological chaperone potential in a cellular system, where they can partially reverse the altered properties of mutated NPM. By most likely stabilizing the hydrophobic core of the C-terminal domain these compounds are able, on one hand, to enhance the stability of the mutants in the cell, which otherwise, given their defective folding, are aggregation-prone. More importantly, their action on mutant NPM appears to allow for the partial recovery of NPM interaction with nucleolar components, thus favoring nucleolar retention of the mutants. These compounds may serve as structural scaffolds that promote proper folding of the mutant NPM and are therefore endowed with interest as therapeutic leads for treatment of AML.

## Methods

### Materials

A subset with the first 2560 compounds of the MyriaScreen Diversity Collection from Sigma Aldrich/TimTec (Newark, DE) was used for screening. The compounds (average purity of 95%) were supplied at 2 mg/mL in 100% DMSO. The hits C1, C2, and C3 (see Table S1) were subsequently ordered from Vitas-M Laboratory Ltd. (Apeldoorn, the Netherlands) (purity > 95% for the different batches), prepared at concentrations of 4 mg/mL in 100% DMSO, and stored at −20 °C. The C-terminal domain of the AML-associated NPM mutant E, corresponding to residues 243-298, > 95% pure, was purchased from Chempeptide Ltd, (Shanghai, China). Cell culture media, antibiotics and fetal bovine serum were obtained from Gibco. Monoclonal antibodies anti-fibrillarin (Clone G-8), anti-Hsp70 (Clone C92F3A5), anti-NPM (clone F82291), and secondary antibodies anti-mouse-HRP and anti-rat-HRP were from SantaCruz Biotechnology (Heidelberg, Germany). Anti-α-tubulin was purchased to Sigma-Aldrich (Madrid, Spain) and anti-GPF to Chromotek (Planegg-Martinsried, Germany). Protease inhibitor cocktail and Xtreme GENE 9 DNA transfection reagent were from Roche (Penzberg, Germany).

### Protein purification

A bacterial expression plasmid^35^ encoding the C-terminal domain (the last 52 residues) of NPM with N-terminal His-GST-tag, removable by TEV protease, was created by PCR, using as template a clone of human NPM kindly provided by Dr. Yanping Zhang^36^ (University of Texas). Full length NPM was produced as previously described^9^. The C-terminal domain was expressed and purified using a similar protocol, with a final gel filtration chromatography step in 25 mM Tris/HCl pH 7.5, 100 mM NaCl, 2 mM DTT, and 5% glycerol. Full-length NPM concentration values throughout the text refer to the pentamer. The integrity of the produced proteins was checked by mass spectrometry (SGIker, University of the Basque Country).

### High-throughput screening

For the initial HTS step, we applied DSF by using the fluorescent dye SYPRO Orange that interacts with the hydrophobic areas of denatured proteins^21^. The experiments were performed in a LightCycler 480 Real-Time PCR System (Roche Applied Science), in a total volume of 25 μL in 384-well microplates (Roche Applied Science) with the C-terminal domain of NPM, which was diluted to 50 μM in 50 mM sodium phosphate buffer, pH 7.5, with 5X SYPRO Orange. Compounds were dissolved in DMSO and then added to the protein and SYPRO Orange solution to a final concentration of 80 μg/mL (corresponding to an averaged compound concentration of 200 μM) and 4% DMSO. Samples were incubated at room temperature for approximately 30 min before loading them into the PCR-instrument. Controls with 4% DMSO were performed on each plate. The unfolding curves where registered from 20 °C to 95 °C at 2 °C/min scan rate. The midpoint melting temperature (*T*_m_), and the corresponding shift relative to the reference (Δ*T*_m_ = *T*_m_−*T*_m,ref_) were calculated for each compound. Compounds that increase the *T*_m_ of NPM C-terminal domain were considered to bind and stabilize the protein^21^.

### Fluorescence spectroscopy

Since Trp residues of NPM locate in the C-terminal domain, their fluorescence properties report conformational information specific to that region also in the full-length protein. NPM (10 µM), wild type or mutant E C-terminal domain (50 µM) in 50 mM sodium phosphate buffer pH 7.5, were mixed with 150 µM C1 compound, previously dissolved in DMSO. Emission intensity of Trp (λ_ex_ and λ_em_ 295 and 350 nm, respectively), was recorded in a QuantaMaster™ 300 fluorimeter (Photon Technology Digital) attached to a Peltier device, using quartz cuvettes with a path length of 0.3 cm, and 5 nm slits. For kinetic experiments, the compound (or control) solution was previously heated at 50 °C, then the protein was added and the time course of change in intensity at 350 nm was recorded.

### Circular Dichroism (CD)

The synthetic peptide corresponding to the C-terminal domain of MutE was resuspended in water to a concentration of 10 mg/ml and stored at −20 °C until used. When required, it was diluted in 50 mM sodium phosphate buffer, pH 7.5. C1 compound, dissolved in ethanol (DMSO interferes in CD), was added to a final concentration of 150 µM. CD spectra were obtained at 20 °C between 200 and 260 nm at a scan rate of 20 nm/min, while ellipticity at 222 nm of full length NPM (10 μM), wild type (50 μM) or mutant E C-terminal domain (70 μM) was recorded between 30 and 85 °C at 1 °C/min. Measurements were performed in a Jasco-810 spectropolarimeter equipped with Peltier temperature control, using a quartz cuvette of 0.1 cm path length.

### Surface plasmon resonance (SPR)

Validation of binding and estimation of the concentration of compound at half maximal binding (*S*_0.5_ ≈ K_D_) were carried out in a Biacore T200 instrument (GE Healthcare, UK). Thereby, a solution of full length NPM (~ 4 μM) in 10 mM sodium acetate pH 4.0 was immobilized onto the surface of a CM5-S sensor chip (GE Healthcare, UK) by the standard amine coupling procedure with PBS containing surfactant P20 (0.05%) as running buffer. The reached immobilization levels of NPM onto the CM5-S sensor chip were around 9,200 response units (RU). The low experimental response levels obtained, which indicate that part of the immobilized protein was not active for the binding (approx. 80%), did not affect in any case the measurements since the different quality control checkpoints were satisfactory: no drifts in baseline and control response, no carry-over between samples, no binding to blank/reference surface. After 1 h baseline equilibration, compounds C1, C2 and C3 were assayed in a concentration-dependent manner (0–500 µM), in running buffer containing 5% DMSO at 25 °C, 30 µL/min flow rate, contact and dissociation time of 60 s and final wash after injection with a 50% DMSO solution. Additional blank immobilization, solvent correction curve and negative control (assay buffer) were included for the analysis of the sensorgrams within the Biacore T200 Evaluation software (version 2.0). For the estimation of *S*_0.5_ and graph representation SigmaPlot 12.5 was used.

### Protein-ligand modeling

Pocket detection analysis was performed on all 20 conformations in the NMR of structure of NPM C-terminal domain^24^ (PDB ID 2LLH) using fpocket^25^ and LigSite^26^. The pockets were ranked based on druggability as computed by the fpocket program. Docking of C1 to all the 20 conformers of the C-terminal domain was performed with Glide (Schrödinger, LLC, New York, NY). The docking modes were ranked based on the Glide score, and the top scoring binding pose was chosen for MD simulation using the Amber package suite^37^ Parameters for the ligand were generated using the general Amber force field^38^, and charges were calculated following the restrained electrostatic potential (RESP) method using Gaussian and Antechamber. All-atom models of the protein−ligand complexes were subsequently prepared with AmberTools, using the corresponding Amber99SB force field^39^. MD simulations were conducted in explicit solvent (TIP3PE water molecules), using a 1 fs time step propagator and Ewald particle mesh with a 10 Å cutoff. Each simulation was run for a total length of 200 ns and analyzed using the cpptraj^40^ and the Bio3d R-package^41^.

Mutant variants of NPM were prepared through homology modelling using Modeller^42^ of 4 representative structures obtained through hierarchical clustering of RMSD based structural similarity^38^ of the 20 NMR structures^24^ (PDB ID 2LLH). 200 ns long MD simulations were performed for each of the resulting mutant models as described for the wild-type simulation above. The resulting trajectories were concatenated and a total of 4000 frames were extracted for pocket analysis using fpocket. The resulting pockets were filtered on distance to pocket #2 and ranked based on druggability score.

### Cell culture, treatment and analysis of cell localization

Cells (HeLa, cervical adenocarcinoma) were cultured as previously described^9^. For subcellular localization analysis of YFP-NPM (wild type and mutants A, E)^9^, cells were transiently transfected with the corresponding plasmid. The compounds (0–150 µM final concentration) were added to the cells 3 hours after transfection, and cultures were maintained for further 24 h. To evaluate the effect of CRM1 inhibition on NPM localization, cells were treated for 3 h with 10 μM leptomycin B (LMB, from Apollo Scientific). After treatments, cells adhered on coverglasses were fixed with 3.7% formaldehyde and mounted onto microscope slides, using Vectashield aqueous mounting medium including DAPI (Vector Laboratories). For immunostaining, fixed cells were permeabilized with 0.2% (w/v) Triton X-100, blocked with 3% BSA and incubated with anti-fibrillarin antibody (1:50 dilution) and anti-mouse-Alexa 594 antibody (1:400 dilution) for 1 h at RT. Slides were examined at the SGIker Analytical and High Resolution Microscopy in Biomedicine Service (UPV/EHU), using a Zeiss Axioscop fluorescence microscope, and photographed with a Zeiss AXIO Observer.Z1 (mode Apotome 2). For semiquantitative assessment of subcellular localization and aggregation of NPM, more than 200 cells were blind-counted per condition, and classified according to two criteria: a) exhibiting predominantly nucleolar YFP-NPM or not, and b) displaying YFP-NPM aggregates or not.

### Toxicity assays

Toxicity was evaluated with the Cytoscan SRB Cytotoxicity assay kit (G-Biosciences) in 96 well plates, titrating HeLa cells (either transfected with YFP-NPM variants or not transfected) with 0–200 μM of compounds C1 and C3. Staurosporine (0–20 μM) was used as control of a toxic compound.

### Immunoblotting

Cells were cultured in 6-well plates, washed with PBS and scrapped in 300 µL RIPA buffer supplemented with protease inhibitors. The cells suspension was incubated with gentle agitation for 30 min at 4 °C, following centrifugation at 10000 xg, 4 °C for 20 min. Supernatant was collected and stored at −20 °C. Proteins were separated in SDS-poliacrylamide gel electrophoresis and transferred to nitrocellulose membranes. Primary anti-HSP70 (dilution 1:200), anti-α-tubulin (1:10000), anti-GFP (1:1000) and anti-NPM (1:400) were incubated overnight at 4 °C in 2.5% (w/v) milk in TBST. Secondary antibodies conjugated to HRP were used at 1:3000 dilution for 1 h, RT. Immunoblot images were recorded in MyECL Imager from Thermo Fisher Scientific and densitometry analysis was performed with Fiji software.

## Electronic supplementary material

Supplementary Information
