# Supplementary material for: Conformational stabilization as a strategy to prevent nucleophosmin mislocalization in leukemia
Source: Sci Rep. 2017 Oct 24;7:13959. doi: 10.1038/s41598-017-14497-4 (PMC5655693; doi:10.1038/s41598-017-14497-4)
Supplement: Supplementary file 1 — Supplementary Information [file 41598_2017_14497_MOESM1_ESM.pdf]

SUPPLEMENTARY INFORMATION

Conformational stabilization as a strategy to prevent nucleophosmin mislocalization in leukemia

María A. Urbaneja[1*], Lars Skjærven[2], Oscar Aubi[2], Jarl Underhaug[2, †], David J. López[1], Igor Arregi[1,‖], Marián Alonso-Mariño[1], Andoni Cuevas[1], José A. Rodríguez[3], Aurora Martinez[2,4*], Sonia Bañuelos[1]

[1]Biofisika Institute (UPV/EHU, CSIC) and Department of Biochemistry and Molecular Biology, University of the Basque Country (UPV/EHU), Leioa, Spain.

[2]Department of Biomedicine and [4]K.G. Jebsen Centre for Neuropsychiatric Disorders, University of Bergen, Norway.

[3]Department of Genetics, Physical Anthropology and Animal Physiology, University of the Basque Country (UPV/EHU), Leioa, Spain.

[†]Present address: Department of Chemistry, University of Bergen, Norway.

[‖]Present address: R&D Department, Roxall España, Bilbao, Spain

*Correspondence: mariaangeles.urbaneja@ehu.es (M.A.U.), aurora.martinez@uib.no (A.M.)

Table S1: Identity, ΔT_m-values, i.e. upshift of midpoint denaturation temperature (T_m) of C-terminal domain of NPM, and MW for the top-three MyriaScreen Diversity Collection members that stabilize NPM C-terminal domain with ΔT_m>17 ºC.

	ID	PubChem ID	ΔT_m (°C)	MW	IUPAC name
1	C1	4748753	19.7	515.40	N-(5-bromopyridin-2-yl)-2-methyl-5-oxo-7-phenyl-4-pyridin-2-yl-4,6,7,8-tetrahydro-1H-quinoline-3-carboxamide
2	C2	5013975	19.6	469.60	2-methyl-N-(5-methylpyridin-2-yl)-4-(3-methylthiophen-2-yl)-5-oxo-7-phenyl-4,6,7,8-tetrahydro-1H-quinoline-3-carboxamide
3	C3	1285033	17.7	459.52	N-(2,3-dihydro-1,4-benzodioxin-6-yl)-2-[3-(4-methylphenyl)-4-oxoquinazolin-2-yl]sulfanylacetamide

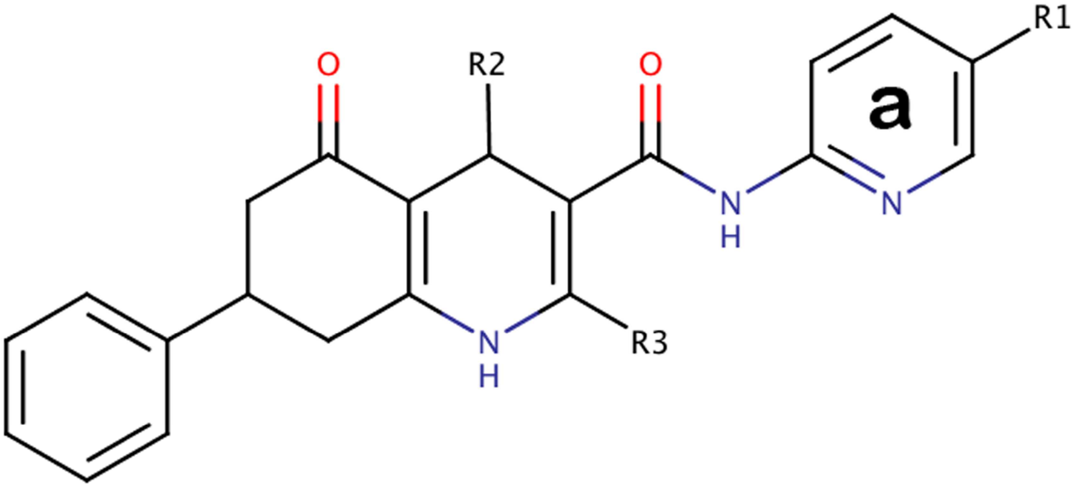

Figure S1. Common scaffold in the chemical structure of compounds C1 and C2; “a” denotes the pyridine ring.

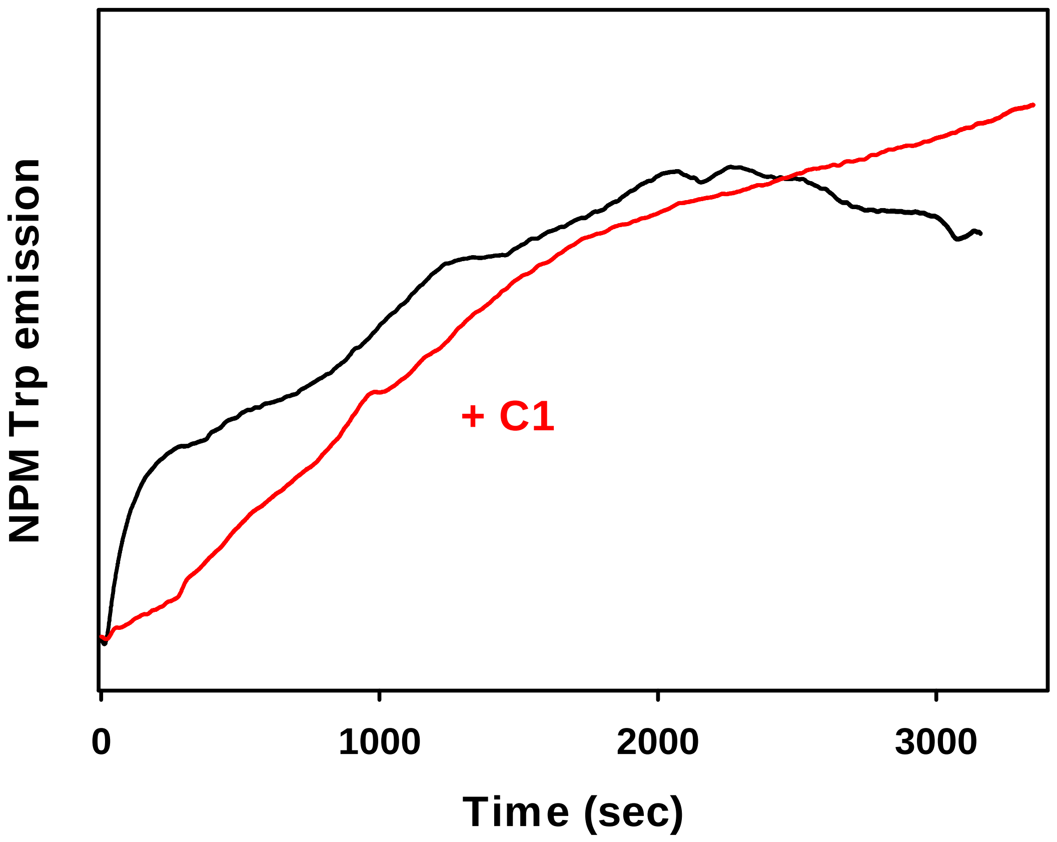

Figure S2. Time course of the denaturation of full length NPM upon isothermal incubation at 50 ºC, in the absence (black) and presence (red) of C1 (50 µM), monitored by the tryptophan fluorescence emission (λ_{ex}= 295 nm and λ_{em}=350 nm).

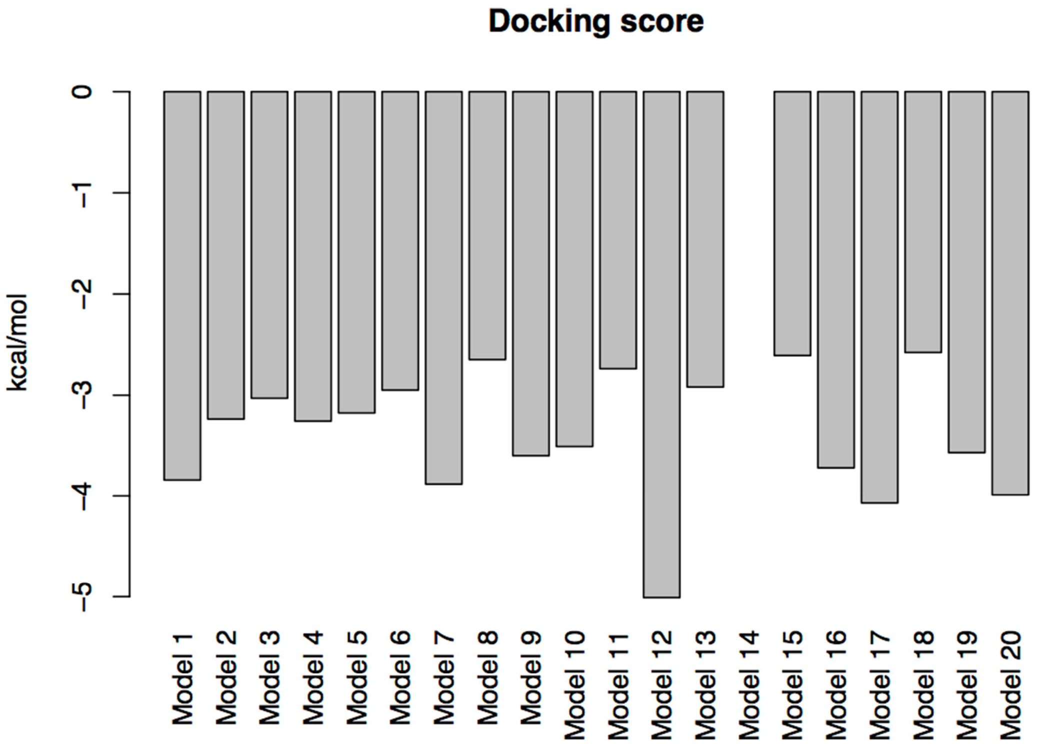

Figure S3. Protein-ligand docking scores. Protein-ligand docking of C1 to all 20 NMR conformations (models) of NPM C-terminal domain (PDB ID 2LLH) shows that docking to model #12 yields the most favorable docking score. C1 could not be docked to Model 14.

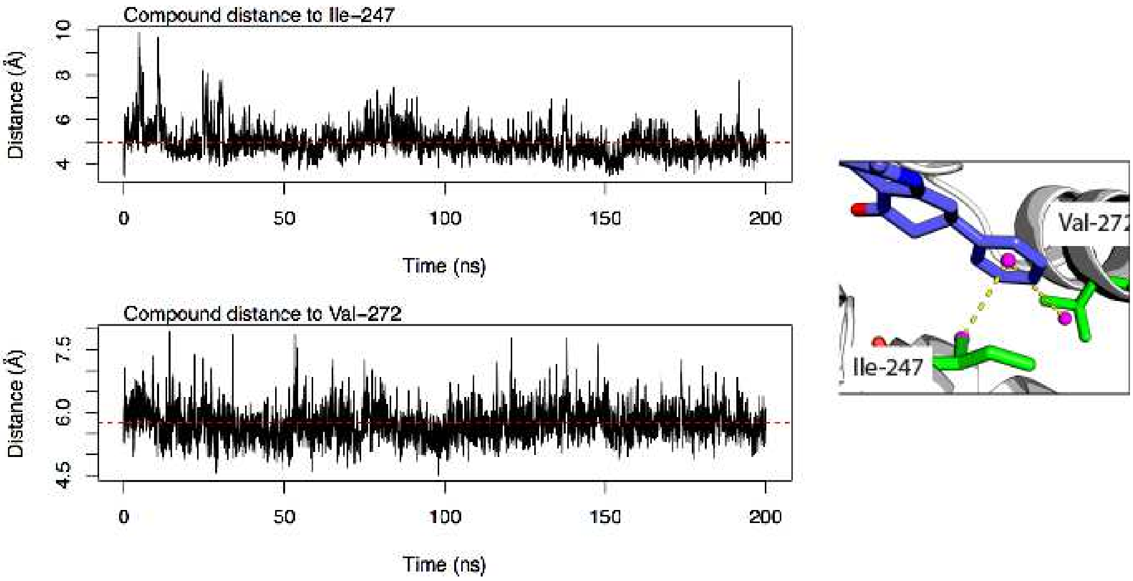

Figure S4. Molecular dynamics simulation of the most favorable docking mode shows stable interactions between core hydrophobic residues and C1 over a 200 ns long simulation. The distances are calculated as the center of mass between the phenyl ring of C1 and CG2 atom of Ile247, and CG1 and CG2 of Val272. The red dashed line depicts the ensemble average.

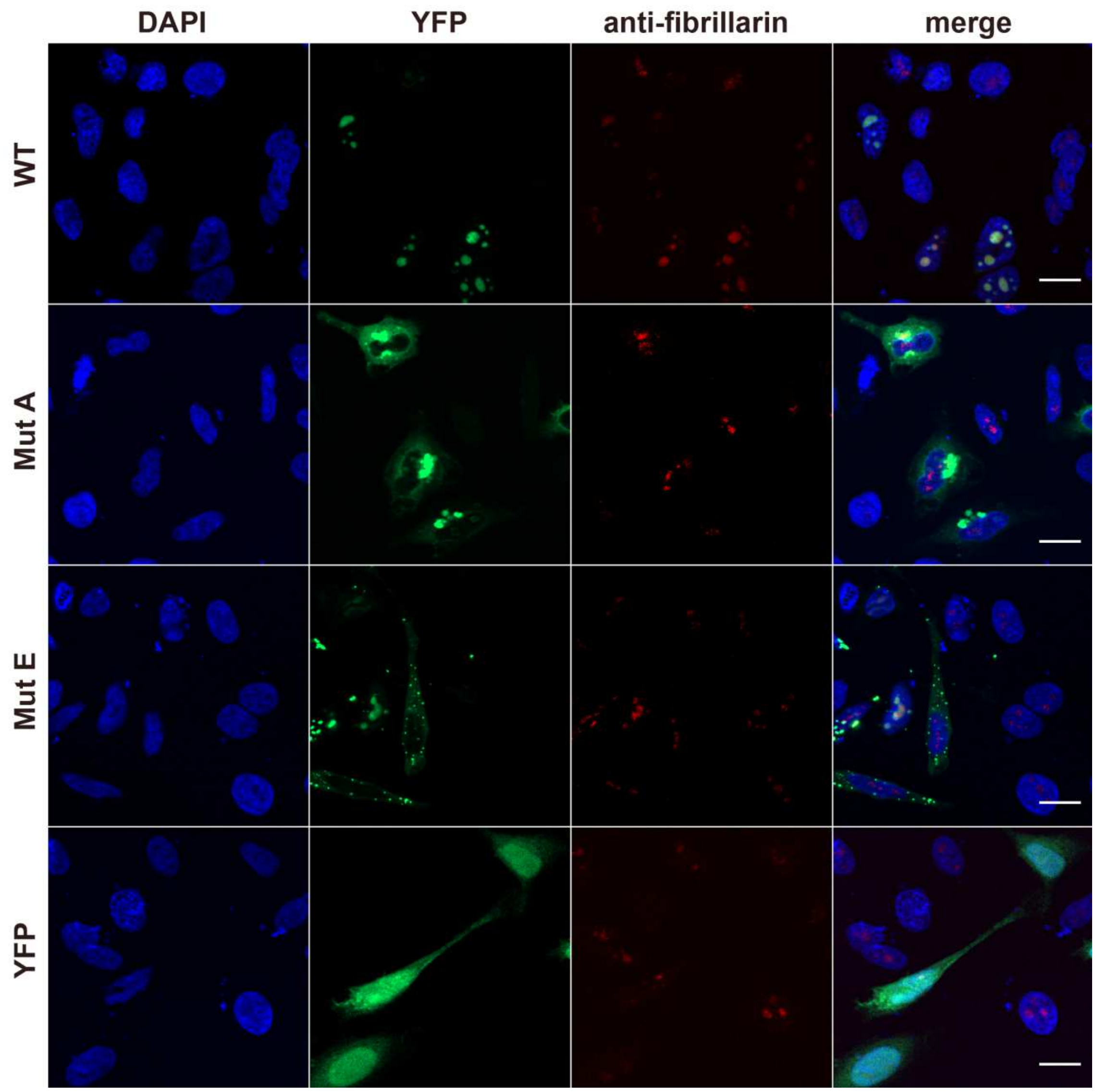

Figure S5. Confocal micrographs of HeLa cells overexpressing YFP-NPMwt (WT), YFP-NPMmutA (MutA), YFP-NPMmutE (MutE) and YFP alone (YFP). Nuclei stained with DAPI are shown in blue. Recombinant YFP-NPM proteins were detected by means of YFP fluorescence (green) and the nucleolar protein fibrillarin was immunostained with anti-fibrillarin monoclonal antibody (SantaCruz, 374022, clone G-8) (red). Cells transfected with YFP-NPMwt showed colocalization of recombinant NPM with fibrillarin (yellow colour in merged images), while YFP signal was mainly detected outside nuclei in other conditions. Scale bar = 20 microns.

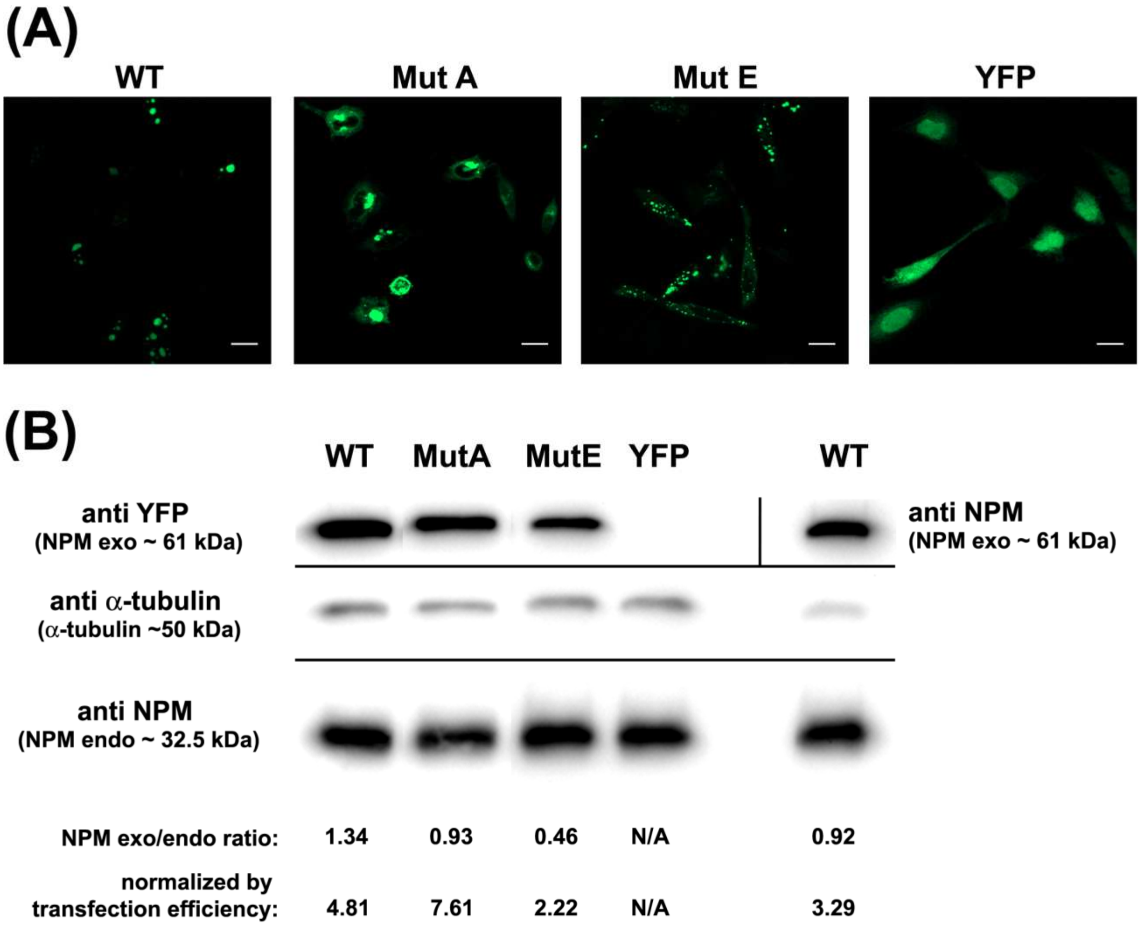

Figure S6. Relation between nucleophosmin levels, transfection efficiency and protein aggregation. (A) Confocal micrographs of HeLa cells transfected with wild type YFP-NPM plasmid (WT), mutant A (Mut A), mutant E (Mut E) and YFP alone. Aggregates were mainly observed in HeLa cells transfected with YFP-NPM mutE and, at a lesser extent, in those transfected with YFP-NPM mutA. Scale bar = 20 microns. (B) Immunoblotting of the corresponding HeLa cell extracts. Endogenous NPM was detected with anti-NPM monoclonal antibody (Santa Cruz, sc-56622, clone FC82291). Exogenous wild type NPM was detected by either anti-GPF monoclonal antibody (Chromotek, 3H9) or anti-NPM, while mutant NPM variants could only be detected by anti-GFP. Densitometric analysis of NPM levels are represented as NPM exo/endo ratio and normalized by the transfection efficiency values for each condition. Transfection percentages were 27.8 ± 0.02% (WT), 12.3 ± 0.03% (Mut A) 20.8 ± 0.01% (Mut E) and 24.8 ± 0.05% (YFP).

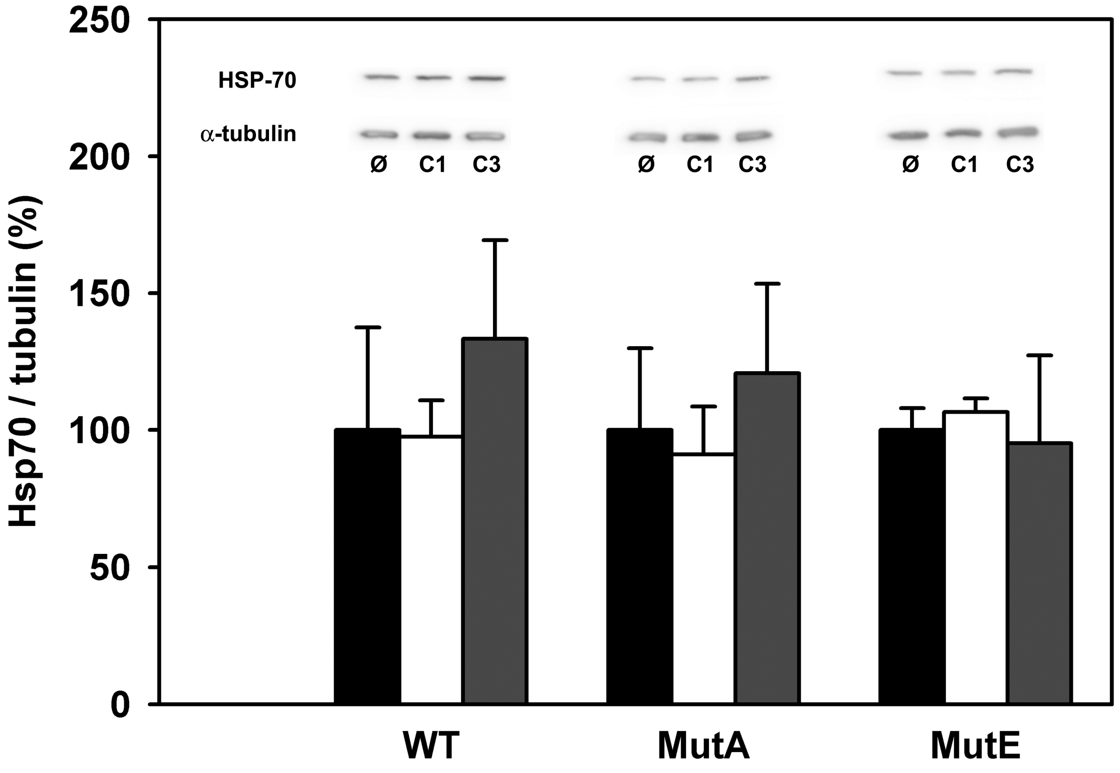

Figure S7. Changes in HSP70 levels after C1 and C3 treatment in HeLa cells. HeLa cells were transfected with either YFP-NPMwt (WT), YFP-NPMmutA (MutA) or YFP-NPMmutE (MutE) and treated with 50 µM C1 (C1, white bars) and 50 µM C3 (C3, grey bars). DMSO was used in non-treatment controls (Ø, dark bars). HSP70 was detected with anti HSP70 monoclonal antibody (Santa Cruz, sc-66048, clone C92F3A-5) and the densitometry values were normalized with α-tubulin. Values of treatments with compounds C1 and C3 were referred to the control (untreated) arbitrarily assigned value. Above each bar, a representative immunoblot image is showed for the corresponding condition.
